# Covalent and Density-Controlled Surface Immobilization of E-Cadherin for Adhesion Force Spectroscopy

**DOI:** 10.1371/journal.pone.0093123

**Published:** 2014-03-27

**Authors:** Dagmar Fichtner, Bärbel Lorenz, Sinem Engin, Christina Deichmann, Marieelen Oelkers, Andreas Janshoff, Andre Menke, Doris Wedlich, Clemens M. Franz

**Affiliations:** 1 Karlsruhe Institute of Technology (KIT), DFG-Center for Functional Nanostructures, Karlsruhe, Germany; 2 University of Göttingen, Institute of Physical Chemistry, Göttingen, Germany; 3 Justus-Liebig-University Gieβen, Molecular Oncology of Solid Tumors, Gieβen, Germany; Swiss Federal Institute of Technology Zurich, Switzerland

## Abstract

E-cadherin is a key cell-cell adhesion molecule but the impact of receptor density and the precise contribution of individual cadherin ectodomains in promoting cell adhesion are only incompletely understood. Investigating these mechanisms would benefit from artificial adhesion substrates carrying different cadherin ectodomains at defined surface density. We therefore developed a quantitative E-cadherin surface immobilization protocol based on the SNAP-tag technique. Extracellular (EC) fragments of E-cadherin fused to the SNAP-tag were covalently bound to self-assembled monolayers (SAM) of thiols carrying benzylguanine (BG) head groups. The adhesive functionality of the different E-cadherin surfaces was then assessed using cell spreading assays and single-cell (SCSF) and single-molecule (SMSF) force spectroscopy. We demonstrate that an E-cadherin construct containing only the first and second outmost EC domain (E1-2) is not sufficient for mediating cell adhesion and yields only low single cadherin-cadherin adhesion forces. In contrast, a construct containing all five EC domains (E1-5) efficiently promotes cell spreading and generates strong single cadherin and cell adhesion forces. By varying the concentration of BG head groups within the SAM we determined a lateral distance of 5–11 nm for optimal E-cadherin functionality. Integrating the results from SCMS and SMSF experiments furthermore demonstrated that the dissolution of E-cadherin adhesion contacts involves a sequential unbinding of individual cadherin receptors rather than the sudden rupture of larger cadherin receptor clusters. Our method of covalent, oriented and density-controlled E-cadherin immobilization thus provides a novel and versatile platform to study molecular mechanisms underlying cadherin-mediated cell adhesion under defined experimental conditions.

## Introduction

E-cadherin is the best-studied member among the calcium-dependent cell-cell adhesion molecules. Homophilic binding between E-cadherins from neighboring cells organizes cells into epithelia which is essential for morphogenetic processes during embryonic and organ development but also for maintaining tissue integrity and homeostasis [1]. The adhesive function of E-cadherin is also required in stem cell renewal [2], [3]. Conversely, loss of the E-cadherin function correlates with tumorigenesis [4], embryonic lethality [5], [6] and loss of pluripotency of embryonic stem cells [7]. To foster firm adhesion the cytoplasmic part of E-cadherin must be linked to the actin cytoskeleton via β- and α-catenin and additionally, these cadherin-catenin complexes become clustered in adherens junctions [8]. Cadherin adhesion has also been reported to participate in mechanosensing by binding to plakoglobin and recruiting keratin filaments to sites of tension at the cell membrane [9].

**Figure 1:**
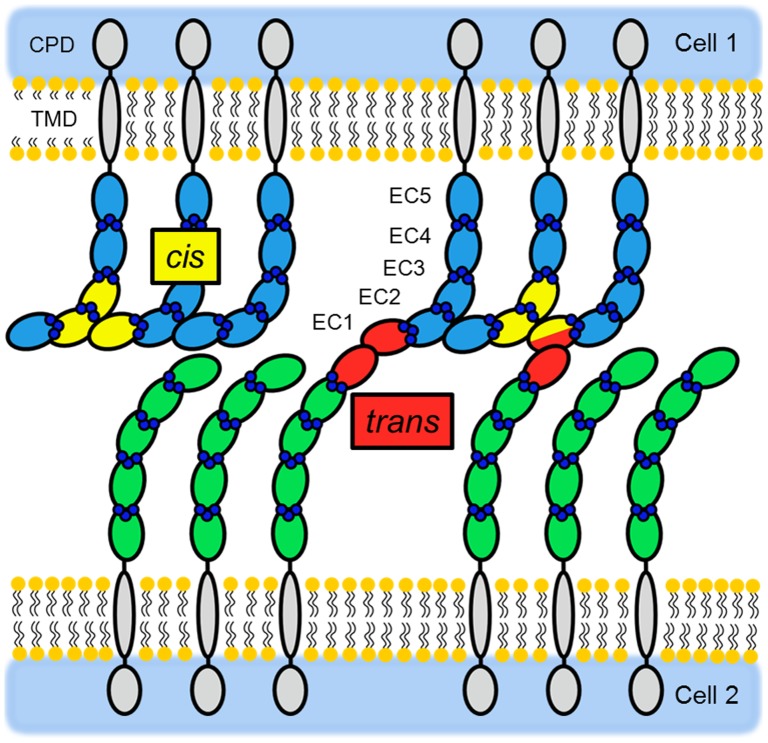
Schematic depiction of *cis*- and *trans*-interactions between E-cadherin ectodomains (ECs) on the cell surface. *Trans*-interactions (highlighted in red) are mediated by the EC1 and occur between cadherin molecules of neighboring cells. Lateral *cis*-interactions (indicated in yellow) occur between the EC1 of one cadherin molecule and the EC2, the EC2/EC3 linker region and part of the EC3 of an adjacent cadherin molecule exposed on the same cell. A combination of *cis*- and *trans*-interactions is depicted on the right hand side of the scheme. Here, the EC1 is simultaneously engaged in a *cis*-interaction with an adjacent cadherin molecule from the same cell, as well as in a *trans*-interaction with a cadherin molecule from an opposite cell (the EC2 involved in both *cis*- and *trans*- interactions is colored in yellow and red). Model based on [14], [57], [58].

E-cadherin is a single-pass transmembrane protein consisting of five extracellular cadherin repeats (ECs). Each EC contains approximately 110 amino acids, which together form a β-barrel structure, while the interface between the ECs contains three calcium binding sites. Calcium binding is required for cell adhesion because it stabilizes the rod-like conformation of the cadherin protein and prevents it from proteolytic degradation [10], [11]. While the cadherin structure has been established, the initial binding mechanisms of cadherins are still a matter of debate. The classical model describes that lateral *cis*-dimerization of cadherin molecules has to take place first before a *trans*-contact with a corresponding dimer of an opposing cell can be formed (experimental evidences are detailed reviewed in [12]). In the last five years, however, improvements in analytical methods have produced results favoring an alternative model in which *cis*-dimerization is no longer critical for *trans*-interaction. Instead, EC1-EC2 interdomains of two cadherins form a fast-binding intermediate *trans*-contact, the so-called X-dimer, which then facilitates formation of more stable swapped *trans*-dimer. In the *trans*-dimer tryptophan 2 (Trp2, W2) of one EC1 inserts into the hydrophobic pocket of its EC1 counterpart, while the backbone of the EC1 domain interacts laterally with the EC2/EC3 domains of an adjacent cadherin forming cadherin *cis*-dimers (**Figure 1**) and, with growing numbers of contributing molecules, a cadherin lattice [13], [14]. In the alternative model cadherin *trans*-interaction does not require pre-formation of a *cis*-dimer. Instead, lateral dimerization and clustering have a cooperative effect by increasing the probability of *trans*-dimer formation [15], [16].

The general significance of lateral cadherin clustering to foster adhesion strength is beyond dispute, but the recent new insight into mechanisms of cadherin binding ask for a re-investigation of the precise effect of clustering on adhesion force. To quantify the clustering effect in adhesion force generation, surfaces carrying cadherin monomers immobilized at defined distances should be provided for cell binding studies. However, many previous cell adhesion studies were based on the classical assumption that *cis*-dimerization precedes the *trans*-interaction of cadherins and therefore used EC domains fused to the heavy chain constant fragment of immunoglobulin (Fc) for surface immobilization [17]. On these artificial surfaces, formation of disulfide bonds between the Fc fragments serves to mimic *cis*-dimerization of cadherin EC domains, which was then predicted to facilitate *trans*-interactions with cellular cadherins. In some of these studies, cadherin-Fc fusion proteins were directly spotted on surfaces for immobilization [18], [19], a procedure which may induce artificial protein folding or surface-induced protein denaturing. Importantly, directly adsorbed fusion protein may also not be in the correct orientation for receptor binding. To circumvent these potential problems, in other studies fusion proteins were immobilized in an oriented manner on surfaces covered with protein A or antibodies recognizing the Fc domain [20]. These non-covalent sandwich strategies potentially provide proper receptor orientation but may not provide quantitative binding. Although cells bind specifically to these cadherin surfaces, studies evaluating the optimal lateral density and geometry of positioning of cadherin EC domains on the surface are still limited.

To overcome current limitations regarding correct receptor positioning, quantitative receptor immobilization and density control, we have applied a SNAP-tag immobilization technique that permits the immobilization of cadherin molecules with controlled intermolecular spacing on patterned surfaces. To this end, different E-cadherin EC domain constructs were fused to a C-terminal SNAP-tag. The SNAP-tag is an enzyme that recognizes benzylguanine (BG) as its substrate and binds covalently to the benzyl group while releasing guanine [21], [22]. For surface immobilization, thiols carrying a BG headgroup can be self-assembled into monolayers (SAMs) on gold surfaces [23]. Upon reaction of the E-cadherin SNAP-tag fusion protein with the BG head group, cadherin becomes covalently attached to the SAM. To control cadherin functionalization density, thiols carrying BG head groups (BGT) are mixed with an unfunctionalized matrixthiol (MT) at different ratios. We confirm the functionality and specificity of the generated E-cadherin-functionalized SAMs in cell spreading assays and by single-cell force spectroscopy (SCFS) and single-molecule force spectroscopy (SMFS). We furthermore demonstrate that E-cadherin constructs containing all five EC domains (E1-5) promote cell spreading as long as a lateral spacing of 5-11 nm is maintained. In contrast, a shorter extracellular fragment of E-cadherin (E1-2) containing only the N-terminal EC1 and part of the EC2 domain fails in cell spreading assays. SMFS confirmed the cell spreading results, as only the E1-5 construct revealed binding forces in the range of 50–70 pN, typical for classical interaction forces, while interaction forces of the truncated construct E1-2 were reduced to near background levels (≤20 pN). Together, these experiments demonstrate that monomeric E-cadherins immobilized via a SNAP-tag provide a suitable tool to study quantitatively mechanism of cadherin clustering in adhesion force generation.

## Methods

### Protein expression and purification

Fusion proteins were generated using the SNAP-vector pSEMS1-26 m (Covalys). Human E-cadherin-EC1-5 or human E-cadherin-EC1-2 fragments were amplified by PCR and inserted via an EcoRV site N-terminally to the SNAP-tag. The 12xHis-tag was amplified by PCR and inserted via Xho/NotI sites C-terminally to the SNAP-tag. A stop codon was added downstream of the His-tag using mutagenesis PCR. HEK293 cells were stably transfected with the different E-cadherin expression constructs. Cells were seeded semiconfluent and cultured for 1 week. Every second to third day the supernatant was collected and protease inhibitors (cOmplete tablet, Roche) were added. Pooled supernatants were filtered and concentrated using VivaCell filtration devices (MWCO 30 kDa, Sartorius). His-tag protein purification was performed using Ni^2+^-NTA column chromatography. Fractions containing the fusion proteins were examined by western blot analysis using an anti-SNAP antibody (NEB) at a dilution of 1:500. Positive fractions were pooled, 1 mM dithiotreitol (DTT) was added and the proteins were stored at −80°C after shock-freezing in liquid nitrogen.

### Surface preparation for cell spreading assays

For cell spreading assays patterned surfaces carrying different E-cadherin EC domain constructs (E1-2 or E1-5) were prepared by microcontact printing (μCP) of BG thiol mixtures on gold as described before [23]. SNAP-tagged E-cadherin EC domains were then covalently bound to the BG headgroups of the thiols forming a SAM on the gold surface. Briefly, a mixture of BG thiol (BGT) and matrixthiol (MT) at a total thiol concentration of 100 μM was incubated on a PDMS stamp for 5 minutes. The dried stamp was then brought in contact with a gold-coated glass cover slip (150 nm gold on 20 nm chromium), removed and the non-patterned areas were back-filled with 100 μM OH-terminated tetra(ethylene glycol)undecanthiol (EG_4_-thiol, Asemblon) for 45 min. Surfaces were washed 3 times with HBS (10 mM HEPES, 150 mM NaCl, pH 7.4) prior to the incubation with 2 μM E-cadherin fusion proteins in HBS for 2 hours at room temperature. After washing three times with cell culture medium, the surfaces were immediately used in cell spreading assays. For SCFS experiments, gold surfaces were incubated with BG-thiol/matrixthiol solutions for at least 16 hours for forming homogeneous SAMs. Afterwards, surfaces were incubated with SNAP-tag fusion protein solution (2 μM) in HBS for 2 hours at room temperature, washed 5 times with HBS and used for force spectroscopy experiments within 1 hour.

### Surface preparation for single-molecule spectroscopy

Cantilevers (BioLever, BL-RC150VB, gold-coated on both sides, Olympus) and gold-coated glass cover slides were cleaned in argon plasma for 20 s and subsequently functionalized by immersion in the thiol solution for 16 h (substrates) or 3 h (cantilevers). Prior to functionalization with cadherin molecules, cantilevers and substrates were rinsed with pure solvent and with EDTA-buffer (2 mM EDTA in HBS) to remove excess thiol from the solution. Both surfaces were simultaneously incubated with protein solution (C_protein_  =  1–2 μM in EDTA-buffer) for 2 h to couple E1-2 or E1-5 to tip and substrate. For heterophilic measurements tips were coated with E1-2 and substrates with E1-5. Prior to force spectroscopy measurements, cadherin-coated surfaces were washed with Ca^2+^-buffer (2 mM Ca^2+^ in HBS) and activated by incubation in the same buffer for 30 min.

### Cell spreading assay and immunochemistry

Untransfected cells or cells stably transfected with human E-cadherin-EGFP were cultured at 7% CO_2_ and 37°C in DMEM high glucose 4,5 g/l (PAA) (L-cells) or in RPMI 1640 (PAA) (HeLa cells). Cell culture media were supplemented with 1% Pen/Strep (PAA), 10% heat inactivated FCS (Biochrom AG) and, in case of the transfected cells, with 2 mg/ml G418 (geneticin sulfate, Roth). For spreading assays cells were washed with PBS (phosphate buffered saline: 137 mM NaCl, 2.7 mM KCl, 6.5 mM Na_2_HPO_4_, 1.5 mM KH_2_PO_4_, pH 7.5) and treated with separation media (2% heat inactivated chicken sera, 2 mM EDTA in PBS). The cells were resuspended in media without other additives and centrifuged. Cells (1.1 × 10^5^ × 2.5 ml) were seeded in a petri dish (35 mm diameter) containing the functionalized surfaces. To verify cadherin-mediated interactions, 2 μl/ml of the E-cadherin blocking antibody DECMA (Sigma-Aldrich) or 10 mM EDTA were added and cells were cultured for 2 hours before being fixed and analyzed by immunostaining. Cells were fixed with 4% paraformaldehyde for 10 minutes. Samples were washed three times with PBS and three times with 2.5% BSA in PBS (blocking reagent) prior to an additional incubation in blocking reagent for 30 min. Antibody incubation (α-E-cadherin, H108, polyclonal rabbit, Santa Cruz, dilution 1:250; α-E-cadherin, DECMA, monoclonal rat, Sigma-Aldrich, dilution 1:500, α-SNAP-tag rabbit polyclonal, ThermoFisher, 1:500) was performed overnight at 4°C or for 1 hour at 37°C. After three washing steps with PBS, secondary goat anti-rabbit-cy3 or goat anti-rat-cy3 antibody (dilution 1:200, Dianova) was applied for 30 minutes at 37°C. For nuclei staining, DAPI (dilution 1:1000 in PBS) was used, followed by three washing steps with PBS. After mounting the samples in Mowiol-488/DABCO (Roth), cells were examined by fluorescence microscopy (Spinning Disc Microscope Cell Observer SD, Carl Zeiss Jena). For statistical evaluation we determined the percentage of spread cells of all cells (spread or rounded-up) attached to the micropatterns (E1-2 or E1-5) or to the passivated EG_4_ area.

### Single-molecule force spectroscopy (SMFS)

Force spectroscopy measurements were performed both in presence and absence of calcium ions. The reversibility of calcium binding was tested by switching from Ca^2+^- to EDTA-buffer and back to the original buffer. Buffer exchange was accomplished by thoroughly rinsing the system with 5 ml of buffer. In order to maintain protein functionality, samples were always kept submerged in buffer. After exchanging the buffer to Ca^2+^- or EDTA-buffer, the system was incubated for 30 min before starting force measurements. The cantilever spring constant (nominal spring constant *k_c_*  =  6 pN/nm) was determined prior to each experiment by the thermal noise method [24]. The force-distance curves were performed with a pulling velocity between 0.1 and 10 μm/s in EDTA- or Ca^2+^-buffer at RT. Contact forces were in the range of 30 to 200 pN and the contact time varied between 0 and 5 s.

### Single-cell force spectroscopy (SCFS)

For SCFS L-cells were transfected with the human E-cadherin-EGFP construct by electroporation. Cells (1×10^7^) were washed twice with 5 ml ice-cold electroporation buffer (120 mM KCl, 10 mM K_2_PO_4_/KH_2_PO_4_, 2 mM MgCl2, 25 mM Hepes, 0.5% Ficoll 400; pH 7.6), resuspended in 300 μl electroporation buffer and transferred into an ice-cold electroporation cuvette (4 mm) containing 10 μg of plasmid. Subsequently, cells were electroporated using a Gene PulserXcell electroporation system (Biorad), an exponential-decay pulse protocol and settings of 250 V and 960 μF. After re-plating, cells were cultured for 16 hours before commencing SCFS measurements. SCFS was performed using a Nanowizard II AFM (JPK Instruments) mounted on top of an Axiovert 200 inverted microscope (Carl Zeiss). A CellHesion module extended the vertical range to 100 μm by piezo-driven movements of the sample holder. Tipless silicon nitride cantilevers were V-shaped, 200 μm long and had a nominal spring constant of *k_c_*  =  60 pN/nm (NP-0, Veeco Instruments). The cantilevers were plasma-cleaned prior to functionalization with concanavalin A as described previously [25]. Cantilever spring constants were determined in situ prior to every experiment, using the microscope's calibration routine, and were found to be compatible with the manufacturer's specifications. Spectroscopy experiments were performed at 37°C using a temperature-controlled BioCell chamber (JPK Instruments). Immediately prior to measurements, cells were washed with D-PBS, treated with separation media, washed and resuspended in fresh CO_2_-independent cell culture medium (Invitrogen). A glass coverslip carrying a gold-coat functionalized with cadherin ectodomain on the left side was inserted into the AFM sample chamber and covered with CO_2_-independent medium. A fraction of the cell suspension was injected into the sample chamber and a single transfected cell, identified by its EGFP signal, was then captured above the transparent right half of the coverslip by pressing the cantilever onto the cell with a contact force of 500 pN for 3 s. The cell was lifted from the surface and allowed to establish firm adhesion on the cantilever for 5 min. To measure surface adhesion, the immobilized cell was lowered onto the functionalized half of the substrate with a contact force of 1.5 nN for different contact times. The cantilever was subsequently retracted at constant speed (5 μm/s) over pulling ranges ensuring complete separation of cell and surface (>70 μm). Usually, two to five force curves were acquired for each cell and contact time interval. Between force measurement cycles the retracted cell was left to recover for 2 to 3 min before being adhered to a different spot on the functionalized surface. Maximal cell detachment forces, single rupture force step height and corresponding loading rates were extracted from retrace curves using the JPK IP Software. To verify cadherin-mediated adhesion, control measurements were performed in the presence of 10 mM EDTA, on EG_4_-thiol SAM lacking the E1-5 construct or by using untransfected L-cells.

## Results and Discussion

### Covalent immobilization of E-cadherin ectodomains

**Figure 2:**
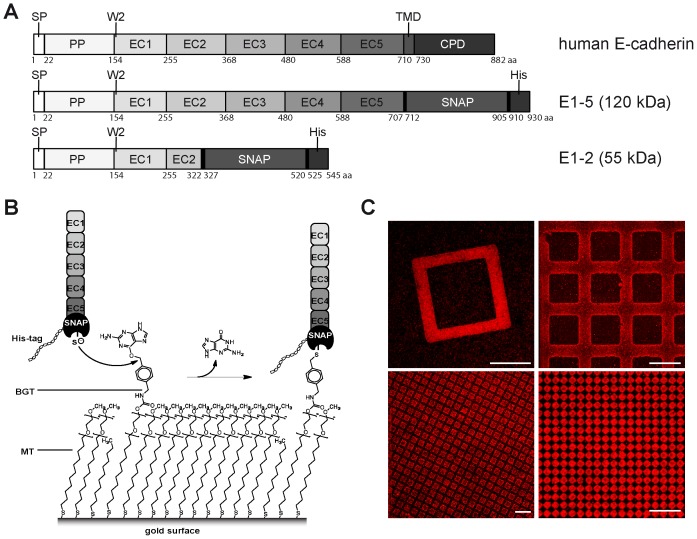
Surface immobilization of E-cadherin constructs. (A) Schematic depiction of the E-cadherin E1-5 and E1-2 fusion constructs fused to SNAP- and His-tag compared to full-length human E-cadherin (upper row). The numbers below the constructs indicate amino acid positions in these constructs. SP: signal peptide, PP: propeptide, EC: extracellular domain, W2: Tryptophan 2, TMD: transmembrane domain, CPD: cytoplasmic domain. (B) Schematic drawing of covalent binding of the fusion protein via its SNAP-tag to the benzyl group by releasing of the guanine part. A mixture of benzylguanine thiol (BGT) and matrixthiol (MT) is applied to the gold surface by microcontact printing (μCP), whereas the unprinted areas are backfilled with EG4-thiol. (C) Immunostaining of the microcontact printed area after protein functionalization using the H108 (top right image) or the DECMA (other images) α-E-cadherin antibody. E1-5 protein binds specifically to the BGT containing areas. Scale bars 20 μm.

We set out to functionalize surfaces with different cadherin EC domain monomers at defined surface density. To this end we generated two different constructs fused to a SNAP-12His-tag in order to make use of the benzylguanine thiol (BGT) self-assembly monolayer (SAM) method we have developed previously [23]. The first construct (E1-5) contains the complete extracellular part of human E-cadherin consisting of five EC domains. The second construct contains a truncated version consisting of the full EC1 and the majority of the EC2 domain (E1-2). Both constructs contain an N-terminal signal peptide and a propeptide required for proper processing of the proteins in eukaryotic cells (**Figure 2A**). While the signal peptide targets the newly synthesized protein to the membrane, the propeptide is thought to protect the N-terminus from premature intracellular cadherin binding [26]. A furin-like protease removes the cadherin propeptide at the cell surface, which limits the production of the cadherin fusion proteins to eukaryotic cell lines, as the protease is absent in bacteria. Of several mammalian cell lines tested, HEK293 cells were the best producers of the E-cadherin-SNAP-12His-tag proteins. A stretch of 12 histidine residues (12His) served to extract the fusion protein out of the cell supernatant by Ni^2+^-NTA affinity chromatography. To loosely mimic the belt-like or punctate cadherin arrangement occurring in adherens junctions, different patterns of thiol SAMs containing a mixture of 1:100 BGT/MT were generated by microcontact printing (μCP) on gold surfaces. After backfilling with pure EG_4_-thiol to passivate the remaining areas on the gold surface, E-cadherin-SNAP-tag fusion proteins were covalently coupled to the BG headgroups via their SNAP-tag (**Figure 2B**). Upright orientation and chemical composition of these thiol SAMs have been previously characterized spectroscopically by XPS (X-ray photoelectron spectroscopy) and PES (photoelectron emission) [27]. Successful immobilization of the E1-5 construct was further confirmed by immunostaining with antibodies specific for the E-cadherin EC domain (**Figure 2C**) or the SNAP enzyme (data not shown).

### E1-5 ectodomains arrayed at intermolecular distances of 5–11 nm promote E-cadherin-mediated cell spreading

**Figure 3:**
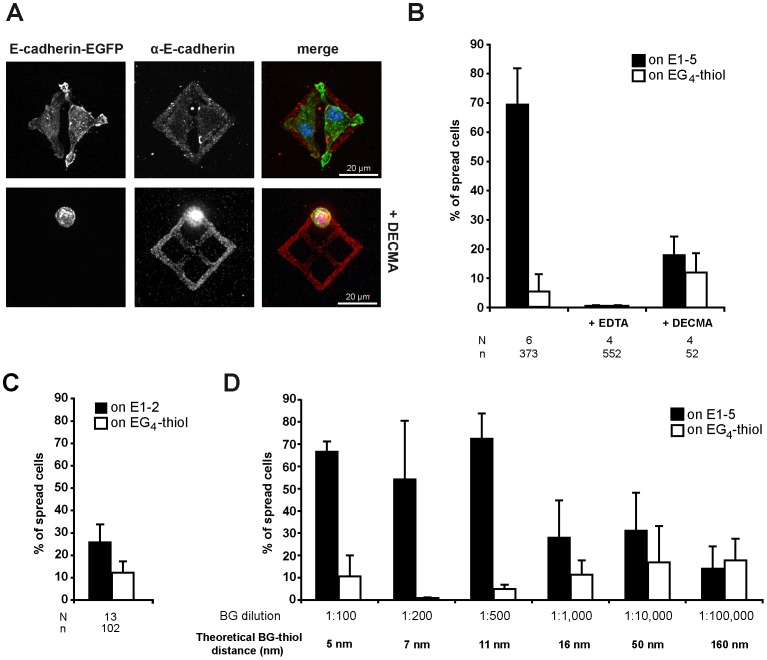
Cell adhesion on immobilized E1-5 and E1-2 fusion constructs. (A) Upper row: fluorescence image of EcadEGFP/L-cells on microcontact printed surfaces functionalized with E1-5; lower row in presence of 2 μl/ml blocking antibody (DECMA). Green: EcadEGFP fluorescence, red: immunostaining against E-cadherin, merge: overlay with nuclei staining (DAPI). (B) Statistical analysis of spread EcadEGFP/L-cells on E1-5 pattern (black) or on EG_4_-thiol (white) without treatment or in the presence of 10 mM EDTA or 2 μl/ml DECMA. Spread cells as percentage of total attached cells per substrate type (mean±SE). N: numbers of experiments, n: numbers of cells. (C) Statistical analysis of spread EcadEGFP/L-cells on E1-2 pattern (black) or on EG_4_-thiol (white). N: numbers of experiments, n: numbers of cells, mean±SE. (D) Statistical analysis of EcadEGFP/cells on E1-5 pattern of different BGT:MT ratios as indicated (mean±SE). The dilutions used to create varying distances of E-cadherin monomers and the corresponding theoretical intermolecular distances are indicated below.

To demonstrate the functionality of the immobilized E-cadherin ectodomains in cell spreading assays, we generated an L-cell line stably expressing human E-cadherin-EGFP (**Figure S1A**). L-cells are murine fibroblasts which lack endogenous cadherins [28] and are therefore widely used as a standard cell line to investigate specific cadherin-mediated cell behavior, comparing the behavior of cadherin transfected and untransfected cells. Transfected L-cells (EcadEGFP/L-cells) revealed proper membrane localization of human E-cadherin-EGFP (**Figure S1B**). When EcadEGFP/L-cells were seeded on E1-5 patterns, they spread and readily adapted to the rectangular pattern shape (**Figure 3A**, top panel). Cadherin-specific binding of the transfected cells to immobilized E1-5 was tested by seeding cells in the presence of EDTA or an inhibitory anti-E-cadherin antibody (DECMA). Both treatments resulted in a loss of pattern recognition and cell spreading of EcadEGFP/L-cells, though occasionally rounded-up cells remained attached to E1-5 pattern after blocking antibody treatment (**Figure 3A**, bottom panel). Statistical evaluation further confirmed the significance of the results. We considered a spread cell morphology as a read-out of cadherin-mediated adhesion and determined the percentage of spread cells (formation of cellular protrusions and irregular cell shape) of all attached cells (sum of spread and rounded-up cells). While 69 ± 12% of EcadEGFP/L-cells spread on E1-5 patterns, only 18 ± 6% of cells were spread in presence of the DECMA antibody. The addition of EDTA completely prevented cell spreading to the E-cadherin patterns or the EG_4_-thiol background. In Ca^2+^-containing medium, however, a small proportion of cell (5-10%) attached to the EG_4_-thiol background (**Figure 3B**).

Although mechanisms of homophilic cadherin binding have been intensively studied, it is still unclear to which extent precisely the different EC domains contribute to cell adhesion [12], [29], [30]. This prompted us to investigate whether a shorter fragment of E-cadherin, consisting only of the outermost EC1 and the majority of the EC2 domains (E1-2, **Figure 2A**) was sufficient for supporting E-cadherin-mediated cell binding. EcadEGFP/L-cells exhibited poor spreading and never fully adapted their shape to the E1-2 rectangles as observed for E1-5 substrates (not shown). Instead, those cells that attached to E1-2 patterns were usually rounded-up, similarly to when cadherin function was blocked by DECMA treatment on E1-5 substrates (**Figure 3A**). The relative frequency of cells spreading on E1-2 patterns (25 ± 8%) was only slightly higher than on EG_4_ passivated areas (12 ± 5%), or DECMA-treated cells on E1-5 (18 ± 6%, compare columns in **Figure 3B,C**). Thus, E1-2 was insufficient to maintain E-cadherin-mediated adhesion. This is of interest in the debate about the role of the EC domains 2 to 5 in cell adhesion. The alternative model by Zhang et al. [15] and reviewed in [16] suggests that cadherin *trans*-dimer formation only involves interacting EC1 domains, while the lateral *cis*-interaction of the EC1 backbone with the EC2, the EC2/EC3 interdomain and part of EC3 is required for subsequent cadherin clustering and lattice formation at the cell membrane. However, the E1-2 construct lacks part of the EC2 and the EC3, and hence cannot support EC1 to EC2/EC3 interaction. Lack of adhesion functionality of E1-2 therefore supports that EC1 to EC2/EC3 binding is required for full binding activity. We therefore conclude that an EC1 swapped *trans*-dimer formation alone is insufficient for cell binding. Only lateral clustering of cadherin molecules may render sufficient mechanical traction forces for cell adhesion. This idea is in line with previous studies performed with a broad set of C-cadherin deletion mutants pointing to the requirement of the EC3 domain in adhesion [31], [32]. These insights into cadherin monomer function also underline the usefulness of the monomer immobilization provided by the SNAP-tag immobilization technique, compared to systems were pre-formed Fc-dimer constructs are used that may bind cooperatively.

To complement the L-cell spreading experiments, we also investigated the adhesion behavior of HeLa cells transfected with EcadEGFP (EcadEGFP/HeLa) as a second cell system. HeLa cells are negative for E-cadherin but express N-cadherin [33] (**Figure 2A**). EcadEGFP/HeLa cells readily spread on immobilized E1-5 and the cellular EcadEGFP distribution reflected the E1-5 surface pattern, pointing to specific recognition of E1-5 by EcadEGFP (**Figure 2C**). In contrast, cell spreading on E1-2 or the passivating EG_4_-thiol was rarely observed (**Figure 2B,C**). Although HeLa cells express endogenous N-cadherin, untransfected HeLa cells did not spread on immobilized E1-5 (not shown). Thus, cross-reactivity between endogenous N-cadherin and immobilized E1-5 appeared negligible, underlining the specificity of E1-5 recognition by EcadEGFP in HeLa cells.

After confirming the functionality of the complete E-cadherin ectodomain (E1-5) immobilized as monomers in cell spreading assays, we became interested in defining the optimal lateral distance between E1-5 molecules required for promoting cell spreading. If lateral clustering between these adhesion molecules is a prerequisite for providing sufficient mechanical traction forces, the intermolecular distance between E1-5 monomers should not exceed a certain limit so that a lattice can still form from adjacent monomers displayed within the SAMs. As covalent binding of the cadherin fusion constructs via the SNAP-tag to BGT/MT occurs at a 1:1 ratio, the cadherin surface density can be systematically adjusted by reducing the BGT content in the SAMs. As shown in **Figure 3D** the BGT/MT mixture could be diluted up to 1:500 without reducing the cell binding capacities of the E1-5 surfaces. However, further dilution of BGT/MT mixture to 1:1000 or above strongly decreased cell binding. We estimated the theoretical density of immobilized E1-5 based on the work of Harder et al., who defined the packing density of a defect-free thiol SAM to be 0.214 nm^2^/thiolate [34]. As a result, a 1:100 BGT/MT SAM would correspond to a distance between single E1-5 of about 5 nm and a 1:500 BGT/MT SAM to a distance of 11 nm (**Figure S3A,B**). Interestingly, a mixture of 1:1000 BGT/MT, which corresponds to an average distance of 16 nm between single E1-5 molecules based on nearest neighbor distribution, lowers the adhesion ability by more than 50% compared to a 1:500 BGT/MT SAM (**Figure 3D**). Thus, our results point to an optimal lateral distance between E1-5 monomers in the range of 5-11 nm. These results are in agreement with the value of 5.7 nm measured for the optimal surface density of Cadherin-11 ectodomains in supported lipid layers required for proper tissue differentiation [35]. Importantly, in contrast to cadherins anchored in lipid layers, E-cadherin ectodomains covalently immobilized on thiol SAMs are fixed in place and cannot move laterally. Thus, in thiol SAM layers the bending capacity of the EC1-5 ectodomain limits lateral clustering. In regard to the lattices model of Harrison et al. [14], this suggests that a distance above 11 nm is too large to allow the interaction of the EC1 domain of the immobilized E1-5 with the EC2/EC3 domain of its lateral neighbor to form a cadherin lattice.

### Dynamic cell adhesion reinforcement during initial cadherin adhesion

**Figure 4:**
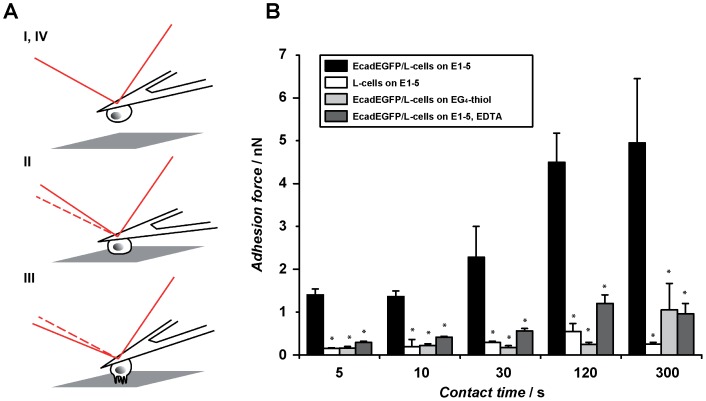
Single-cell force spectroscopy. (A) Schematic depiction of an SCFS experiment. A single probe cell is attached to an AFM cantilever (I) and brought in contact with the substrate (II). During cell retraction (III) a force curve is recorded from which the cell adhesion force can be determined. (B) Adhesion forces (mean ± SEM) measured at different contact times. EcadEGFP/L-cells on E1-5 (black), on EG_4_-thiol (light grey), on E1-5 in the presence of 10 mM EDTA (dark grey) and untransfected L-cells on E1-5 (white) were tested. The significance was determined using the Mann-Whitney test (* p< 0.05). At least 10 cells were tested per condition.

After having determined the optimal density of E1-5 immobilization for overall cell spreading, we proceeded to investigate the effect of receptor density on the formation of cadherin-mediated cell adhesion forces directly by AFM-based single-cell force spectroscopy (SCFS). SCFS provides excellent control over cell/substrate contact times so that the dynamics of cell adhesion formation can be studied. Single EcadEGFP-transfected L-cells immobilized on an AFM cantilever (**Figure 4A**) were approached for different time intervals onto homogeneous E1-5-surfaces produced by using a 1:100 BGT/MT ratio (estimated intermolecular spacing: 5 nm). Subsequently, cell-substrate rupture forces were determined by pulling the cell away from the substrate through an upward movement of the cantilever. As shown in **Figure 4B**, EcadEGFP/L-cells showed progressive adhesion strengthening with increasing contact time, typical for the formation of receptor-mediated cell adhesion. To verify specific cadherin-mediated adhesion, we performed a series of control experiments. On EG_4_-thiol substrates lacking the E1-5 functionalization, adhesion forces were significantly reduced at time points. Likewise, adhesion was significantly reduced when removing extracellular Ca^2+^ by EDTA addition to the medium or when testing untransfected, cadherin-negative L-cells. Together, the control experiments thus confirmed specific, E-cadherin-mediated cell adhesion to the E1-5 substrate and underlined the suitability of these surfaces to study molecular mechanisms of cadherin-mediated adhesion processes.

Over the first 300 s of substrate contact, the build-up of cadherin-mediated cell adhesion forces followed a sigmoidal pattern: Initially (≤10 s), cells showed a low adhesion force of about 1.7 nN, which strengthened about 2.7-fold to 4.6 nN between 30 and 120 s of cell attachment. In the remaining 180 s of contact, only a slight further force increase of less than 10% (from 4.6 to 5 nN) occurred. The formation dynamics and range of cadherin-mediated adhesion forces we measured were on a similar scale as other cadherin-dependent forces reported in several previous studies in different cell types and techniques. For instance, the mean value of traction forces of C2 mouse myogenic cells on N-cadherin coated pillars measured over 20 min is about 15 nN [36]. Measurements of binding probability of cadherins over shorter time intervals were performed by Chien et al. using cell aspiration assays between C-cadherin expressing cells and C-Cadherin functionalized red blood cells [32]. Interestingly, the binding probability increased strongly within the first 2 s, followed by a lag phase between 2-7 s, and again increased until after 20 s a plateau was reached [32]. Using a different approach, we observed a similar biphasic response, although the dynamics of adhesion formation was slightly shifted in SCFS experiments. Between 5 to 10 s we measured a moderate constant adhesion force, while a continuous increase of the adhesion force was observed after 30 s contact time. We assume that the delayed increase in adhesion force generation reflects the input of E-cadherin clustering and cytoskeleton anchorage. A comparable biphasic displacement behavior of N-cadherin loaded beads on myogenic C2 cells has been previously reported [37]. N-cadherin loaded beads showed an initially freely diffusive phase (20–100 s, depending on the density of loaded N-cadherin), followed by a second phase of directed diffusion due to the anchorage of the cellular N-cadherin receptors to the cytoskeleton.

The dynamic reinforcement of cadherin-mediated adhesion forces has not been quantitated before in living cells over time scales extending to several minutes. The forces measured with our method likely reflect the progressive recruitment process of cadherins into new cell-cell contacts, processes which have previously been traced by photobleaching and recovery of E-cadherin-EGFP [38]. Lateral clustering of E-cadherin into cadherin lattices [39] and an increase in the number of engaged receptors in the contact area may lead to the strong force increase, starting 30 seconds after contact initiation. This time window corresponds to E-cadherin puncta formation connected to the actin cytoskeleton at newly formed cell contacts [38]. Subsequently, fusion of the early puncta into larger patches further promotes cadherin clustering, occurring 10–15 min after cadherin-cadherin engagement at the earliest [40]. These later-stage processes of cadherin aggregation were not investigated here. However, the SCFS measurements confirmed the suitability of the SNAP-tag immobilization method to quantitate early events of cadherin-mediated cell adhesion.

### Single-molecule adhesion forces between cadherin monomers

**Figure 5:**
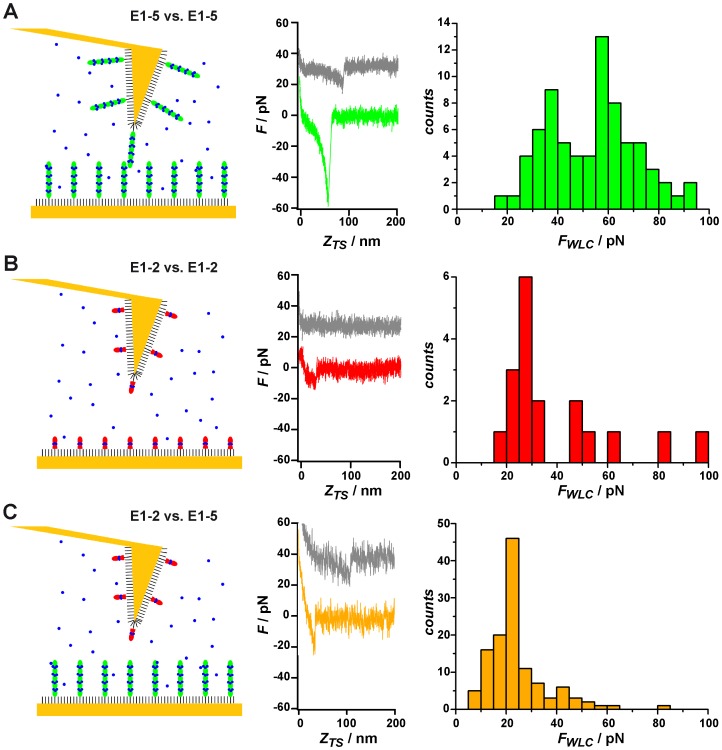
Single-molecule force spectroscopy. (A) Force spectroscopy data obtained during the bond rupture between E1-5 constructs attached to a gold-coated AFM tip and a gold-covered glass slide (see scheme). A typical force extension curve shows the unbinding of E1-5 dimers in the absence of calcium ions (grey) and the presence of calcium ions (green). The extension curves show nonlinear stretching behavior preceding bond rupture. The rupture force histogram (green) reveals two major maxima potentially corresponding to two types of bonds. (B) Corresponding SMFS data of force-assisted unbinding of E1-2/E1-2 (red) or (C) E1-5/E1-2 (orange) cadherin molecules attached to tip and substrate as illustrated in the schemes. Tip substrate contact time was 0.1 s and pulling velocity was 1 μm/s.

After quantitating cadherin-mediated adhesion forces to immobilized E1-5 on the single cell level, we were interested in obtaining further insight into cadherin adhesion forces on the single receptor level. Therefore, we performed single-molecule force spectroscopy (SMFS) experiments with the E1-2 and E1-5 cadherin constructs, subjecting them to homomeric (E1-5/E1-5 or E1-2/E1-2) or heteromeric (E1-5/E1-2) bond breakage under a linear force ramp (**Figure 5A-C**). In these experiments, both AFM-tips and gold surfaces were functionalized with the corresponding cadherin molecules and brought into contact for a defined time, allowing the molecules to form an adhesive complex. Upon withdrawal of the tip from the surface, bond breakage becomes visible as a jump in the retraction curve. When using short contact times (<5 s) and low contact forces (<200 pN), about 20% of all force curves involving E1-5 homodimers displayed unbinding events, while heterodimers composed of E1-2 and E1-5 produced a 8–10% yield under identical contact conditions. In the case of E1-2 attached to tip and substrate, only 5% of all curves displayed rupturing of bonds. The low interaction probability in all experiments indicated that primarily single molecule rupture events were detected according to Poisson statistics. To further confirm that these experiments detected the unbinding of individual bonds, the rupture events were wormlike chain (WLC)-fitted, which provided persistence lengths of *l_p_*  =  (0.5 ± 0.1) nm, indicative of single polypeptide chain stretching (**Figure S4**). Counting only rupture events consistent with single polymer chain stretching (WLC behavior) restricted our analysis to events corresponding to single-molecule mechanics and excluded non-specific contact mechanics dominated by attractive van-der-Waals forces. As a specificity control for cadherin-mediated binding, force curves were collected in the presence and absence of Ca^2+^-ions.

Single-molecule rupture events were recorded as a function of construct size, contact time, presence of calcium ions, and loading rate. **Figure 5A** shows typical force extension curves obtained from disjoining E1-5 homodimers (left column) in the presence (green) and absence (grey) of Ca^2+^ -ions (2 mM). Generally, unbinding forces of homomeric E1-5-bonds were substantially larger in the presence of Ca^2+^ (35–90 pN) than in the absence of Ca^2+^ (0–20 pN) at an identical pulling speed of 1 μm/s. The rupture force histograms for homomeric unbinding of E1-5 displayed two distinct peaks, centered around 35 and 60 pN (**Figure 5A**). While addition of 2 mM EDTA fully abolished specific interactions between E1-5 cadherin ectodomains, a smaller relative impact of Ca^2+^-depletion was found on rupture forces during disjoining of E1-2 dimers (**Figure 5B**, red curve with calcium, grey curve after calcium depletion). However, in this case unbinding forces were already lower (around 20–30 pN, see red histogram in **Figure 5B**). The unbinding of heteromeric E1-2/E1-5 (**Figure 5C**) showed a complex distribution of rupture forces (yellow histogram in **Figure 5C**), but with a lower number of events than observed for the homomeric E1-5 interaction.

**Figure 6:**
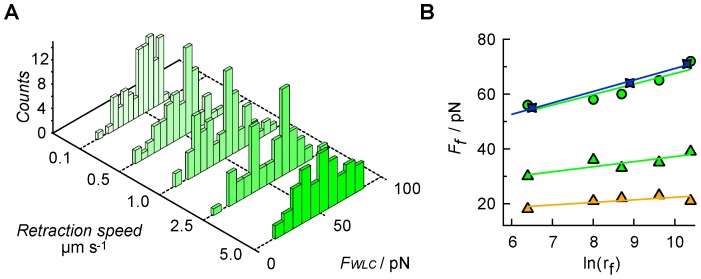
Dynamic force spectroscopy. (A) Rupture force distributions obtained at different retraction speeds for the homotypic E1-5 bond rupture. (B) Rupture force *F*_f_ as a function of loading rate for homotypic E1-5 (green symbols) or heteromeric E1-2/E1-5 bond rupture (orange symbols). The different symbols represent the maxima identified in the multimodal distribution of rupture force obtained for a given loading rate. For the homotypic E1-5 bond we determined x_u_  =  1.1 nm and k_off_  =  1.2 × 10^−4^ s^−1^ (green circles); x_u_  =  2.3 nm and k_off_  =  1.4 × 10^−5^ s^−1^ (green triangles). For the heteromeric E1-5/E1-2 bond we determined x_u_  =  4.5 nm and k_off_  =  5.9 × 10^−7^ s^−1^ (yellow triangles). The violet stars indicate data taken from [41].

To obtain further insight into the cadherin unbinding mechanism, we investigated the loading rate dependency of the unbinding forces (**Figure 6** **)**. Again, only rupture events that displayed clear WLC behavior prior to disjoining were taken into account, excluding non–specific interactions from the analysis. However, based on this requirement, homomeric interactions between the shorter E1-2 constructs could not reliably analyzed due to difficulties in distinguishing between specific and non-specific interactions. Plotting the most probable rupture force *F_f_* as a function of loading rate *r_f_* provides a means to compute the off-rate at zero force *k_off_* (*F*  =  0) and the distance from the ground state to the transition state (*x_u_*) according to equation (1) and assuming a single well potential:
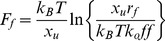
(1)


The E1-5 bond rupture force histograms revealed a bimodal distribution at all tested pulling speeds (**Figure 6A**). We interpreted the bimodal distribution of the homomeric E1-5 bond as the probing of two different types of bonds. Therefore, we plotted the maxima of the first peak (green triangles) at low force and the second peak (green circles) at high force separately over the corresponding loading rates (**Figure 6B**). For comparison, we also added data obtained by Leckband and coworkers (purple stars, [41]), which largely matched our results regarding the stronger bond fraction. Also in agreement with previous data presented by Leckband and coworkers, the width of the potential-well amounted to *x_u_* ∼ 1.1 nm. In contrast to the homotypic E1-5 interaction, the force histograms obtained from E1-2/E1-5 interactions (orange symbols in **Figure 6B**) and E1-2/E1-2 interactions (data not shown) displayed substantially lower forces. However, occasionally we also observed larger forces for these interactions, possibly reproducing the low and high force peaks found for homotypic E1-5 bond breakage.

A bimodal distribution of unbinding forces could be explained either by occasional formation of E1-5 *cis*-dimers prior to *trans*-binding, yielding two different classes of *trans*-interactions depending on the *cis*-dimerization state. Replacing E1-5 by E1-2, which lacks part of EC2, the EC2/3 interdomain region and EC3 important for *cis*-dimerization, resulted in an overall reduction of binding strength, indicating an important role of lateral interactions for modulating the binding strength of E-cadherin. However, *cis*-dimerization alone is unlikely to account for the switch between high and low binding strength in the E1-5 construct and alternative mechanisms may account for the rupture force variability. For instance, different *trans*-interaction sites could exist on a single cadherin strand, which are exposed depending on the precise molecular orientation. Biomembrane force probe measurements also indicate that multiple binding states are responsible for the adhesive contact between cadherins: Leckband and coworkers found two weak bonds with a *k_off1_*  =  3.9 s^−1^ and *k_off2_*  =  0.019 s^−1^ when rupturing EC1–EC2 fragments [41], while EC1–EC5 fragments exhibited four different bond classes differing in strength and off-rate. These different states did not correspond to multiple cross-links, as the dominant peak at higher forces displayed an off-rate (*k_off_*  =  1.2 × 10^−4^ s^−1^) which was similar to our findings for the high rupture force population in single molecule unbinding events.

Zhang and coworkers measured an identical binding strength of wildtype cadherins acting either as monomers or as laterally connected dimers (peak force of 64 ± 27 pN) [15]. Although in these experiments the bond strength of monomeric and cadherin–Fc dimer complexes were similar, the cadherin-Fc dimer showed a higher probability of binding than the cadherin monomer [15], indicating that dimerization may enhance the chance of cadherin binding initiation. In agreement, varying the contact time between the functionalized AFM-tip (E1-5) and the substrate (E1-5) between 0 to 5 s also revealed an increase in binding probability with contact time but no significant impact on the scale of unbinding forces in our measurements. Our single-molecule measurements therefore confirmed that E-cadherins-SNAP-tag fusion constructs display essentially identical binding mechanisms as Fc constructs. Cadherin-SNAP tag constructs therefore provide an alternative system to study cadherin-mediated adhesion processes, albeit with the added benefit of covalent, oriented and density-controlled immobilization.

### Integrating SMSF and SCFS reveals sequential unbinding of cadherin bonds

**Figure 7:**
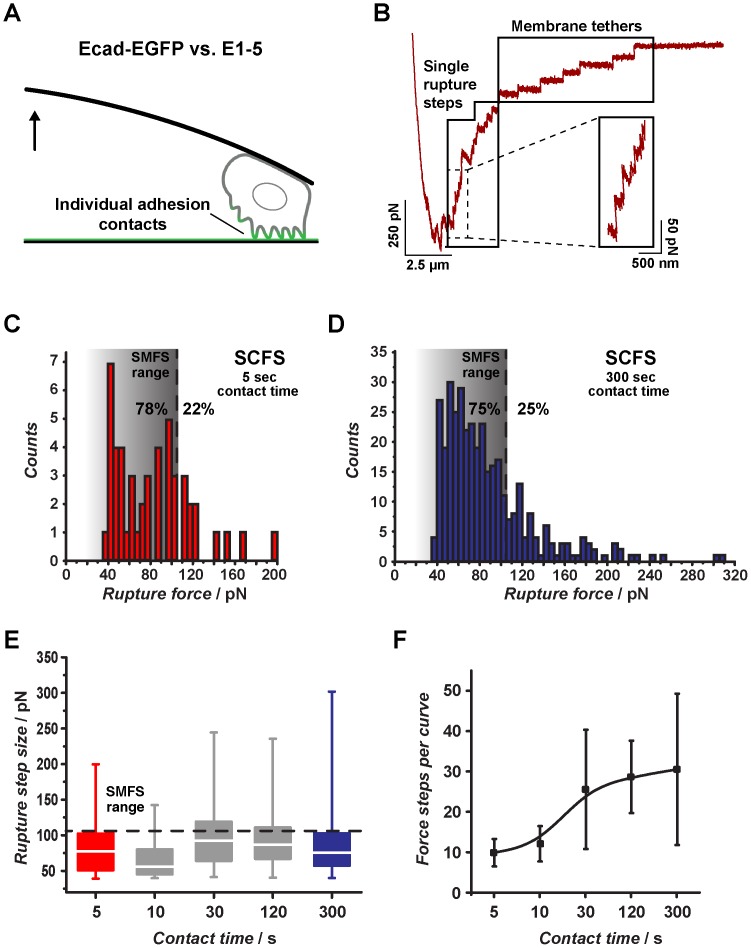
Rupture force step analysis in SCFS experiments. (A) A single L-cell expressing Ecad-EGFP is approached onto an E1-5 surface. During substrate contact individual cadherin adhesion contacts form progressively. (B) During subsequent cell retraction, individual contacts rupture sequentially, leading to a step-like signature in the force-distance curve. Cytoskeleton-attached receptor bonds rupture under active force loading (single rupture steps, zoom-in), while membrane tethers are extracted under constant force, indicted by a plateau in the force curve before bond rupture. A plateau length cut-off of 300 nm was used to separate single rupture steps from tethers. (C) Single rupture step histogram after a contact time of 5 s. The dotted line indicates an arbitrary force threshold which contains <95% of single cadherin unbinding forces determined in SMFS measurements. The percentage values indicate the fraction of single rupture steps falling below (on the right) or above (on the left) the single-molecule force threshold. (D) Single rupture step histogram after 300 s. The percentage values indicate the fraction of single rupture steps falling below (on the right) or above (on the left) the single-molecule force threshold. (E) Rupture step sizes versus contact time as box-whisker-plots. The dotted lines indicate the same single molecule force threshold as in (C) and (D). (F) Number of single rupture steps (±SD) per force-distance-curve for different cell-substrate contact times. A trend line highlights the increase in rupture force step number per force curve with increasing contact time.

As demonstrated above, in SCFS the dynamic formation of cadherin-mediated cell adhesion can be monitored as an increase in cell detachment forces with increasing cell-substrate contact time (**Figure 4B**). However, in addition to quantitative information about overall cell adhesion (maximal detachment force), SCFS force-distance curves also contain a series of discrete rupture force steps which can provide information about the scale and number of individual adhesive contacts that have formed during substrate contact (**Figure 7A**). Analyzing these small rupture steps, which may correspond to the rupture of single receptor bonds or to small groups of receptors unbinding collectively, can provide additional insight into the receptor binding mechanism [42], [43]. For instance, in integrin-mediated adhesion, progressive receptor clustering and adhesion reinforcement coincides with a gradual increase of the single rupture step size above the single-receptor level [42], [44]. However, rupture events group into two mechanistically different classes which must be distinguished during force curve analysis [45]. Single rupture events, or „force jumps“, denote bond rupture of cytoskeleton-associated receptors. Cytoskeletal anchoring stiffens the adhesive bridge and causes a non-linear force increase prior to bond rupture [46]. Since these bonds rupture under force loading, the corresponding rupture force steps can be analyzed to determine the receptor-ligand bond strength in relation to the applied loading rate [43], [47]. In contrast, tethers are small cytoskeleton-free membrane tubes extracted from a large plasma membrane reservoir under constant force, indicated by a force plateau before rupture [48], [49], [50]. Tether rupture forces are related to the force required for membrane tube extraction, but provide no information regarding the strength of individual receptor-ligand bonds at the tether tip or the number of receptors maintaining the tether.

In addition to the slope of the force curve preceding rupture, single rupture steps and tether events can be distinguished by the position within the F-D curve at which they occur [51]. Single rupture steps occur early during retraction while the cell body is detaching from the substrate, whereas tethers are easily extended to several tens of micrometer [52], [53] and often rupture only in the final phase of cell retraction after the main cell body has already detached fully from the surface [45], [51]. SCFS retraction curves on E1-5 substrates contained a characteristic rupture step pattern consistent with the presence of both single rupture steps in the early phase of retraction and tether rupture at lager stages (**Figure 7B**). The tether rupture events showed a regular staircase-like pattern, similar to what has been recently observed for integrin receptors whose intracellular link to the actin cytoskeleton has been severed [54]. To restrict the rupture force analysis to single rupture steps, we defined a plateau length exceeding 300 nm before bond rupture as a criterion for tether behavior [49] and removed all rupture events meeting this condition (23–54% of all rupture events, depending on the cell-substrate contact time) from subsequent rupture force analysis. Quantification of the remaining single rupture steps after 5 s of substrate contact yielded a rupture force distribution spanning from about 35 to 200 pN, with a maximum around 50 pN (**Figure 7C**). At the same retraction speed (5 μm/s), SMFS experiments yielded higher bond loading rates compared to SCFS measurements, indicating a stiffer linker/receptor bridge in the cell-free system, and therefore accelerated force loading before bond rupture. However, at low retraction speed (0.1 μm/s), the mean loading rates of single cadherin unbinding events in SMFS and single rupture steps in SCFS experiments were comparable (∼700 pN/s). Under these conditions, SMFS yielded single cadherin unbinding forces between 15 and 70 pN, with a maxima around 55 pN (**Figure 6**). Thus, at short contact times the majority of single rupture steps in cells were within a similar range as single E1-5/E1-5 interactions measured in SMFS experiments, suggesting that they primarily represent single cadherin-cadherin bond breakage.

To investigate the development of single rupture steps with increasing contact time, we defined an arbitrary upper single-molecule force limit of 105 pN. This cut-off force covered all single cadherin unbinding forces determined at comparable loading rates in SMFS experiments and also the majority of single E-cadherin unbinding forces measured by Panorchan et al. in a CHO cell system at the same cell retraction speed (5 μm/s) [55]. Using this value as an approximate upper force threshold for single-molecule events indicated that the majority of rupture events (>78%) in cells falls within the single-cadherin range at short contact time (**Figure 7C**). After 300 s contact, there is a slight increase in the percentage of single rupture forces ranging above the single cadherin bond threshold, suggesting a partial change from single to cooperative receptor binding or an increase in the adhesion strength of individual receptors (**Figure 7D**). However, the majority of single rupture events (75%) still rang at the single cadherin level across the entire 300 s interval (**Figure 7E**), although overall adhesion increases greatly over the same interval (see **Figure 4B**). This indicated that during cell detachment individual cadherin receptors unbind sequentially, instead of simultaneously as larger groups of cross-linked receptors. However, the number of rupture force steps per force curve increased with contact time, demonstrating a progressive increase in the total number of engaged cadherin receptors with time, which would account for the increase in overall cell adhesion with time (**Figure 7F**). Together, these findings suggest that cadherin contacts are primarily reinforced by increasing the number of engaged receptors and by organizing these receptors into regular yet comparatively loosely associated adhesive clusters. Integrating results obtained from both SMFS and SCFS measurements on structurally and chemically well-defined cadherin adhesion substrates can therefore provide a better understanding of mechanisms underlying cadherin binding in the cellular context.

## Conclusions

In this work we have presented a novel method for covalent surface immobilization of different monomeric E-cadherin constructs using a SNAP-tag approach. This method provides an important alternative to Fc-fusion protein immobilization, and generates patterned cadherin surfaces with defined receptor orientation and adjustable lateral density. We confirmed the biological functionality of these surfaces in cell spreading and AFM single-cell and single-molecule adhesion experiments and determined a receptor spacing of 5 to 11 nm for optimal cell spreading. Furthermore, by integrating SMSF and SCFS measurements we gained additional insight into the formation and rupture mechanics of cadherin bonds and the contribution of individual cadherin ectodomains for mediating cell adhesion. Recently, the SNAP-tag has also been applied to functionalize 3D polymer scaffolds with high special accuracy [56]. When using cadherin SNAP-tag proteins in these applications, the SNAP-tag immobilization method should foster activities aimed at rebuilding organotypic cell assemblies 3D, providing a unique opportunity to investigate cadherin function beyond 2D surfaces.

## Supporting Information

Figure S1 **Western-blot analysis of L-cells.** (A) Western-blot analysis of wildtype L-cells and EcadEGFP expressing L-cells. Merged phase contrast and fluorescence images of EcadEGFP/L-cells (B). Junctional localization of EcadEGFP indicates proper cell adhesion function of the construct in L-cells.
(TIF)

Figure S2 **Western-blot analysis of HeLa cells.** (A) Western-blot analysis of wildtype HeLa cells and EcadEGFP-transfected HeLa cells for E-cadherin (right panel) or N-cadherin (left panel). Statistical analysis of EcadEGFP/HeLa cells spread on E1-5 or E1-2 patterns or on EG_4_-thiol. N: numbers of experiments, n: numbers of cells, standard error is shown (B). Fluorescence image of EcadEGFP/HeLa cells on microcontact printed surfaces functionalized with E1-5 or E1-2. Green (C): EcadEGFP fluorescence, red: immunostaining against E-cadherin (E1-5) or SNAP-tag (E1-2), merge: overlay with nuclei staining (DAPI).
(TIF)

Figure S3 **Average distance calculation of E-cadherin monomers.** (A) Theoretical hexagon grid used for the calculations. Each hexagon marks the area occupied by one benzylguanine thiol diluted in matrixthiol. The intermolecular distance *d* corresponds to 2× the radius *r* of the hexagon. Based on Harder et al. 1998, the area *A* occupied per thiolate is 0.214 nm^2^ [34]. When this value is multiplied with the dilution ratio, the hexagon radius and the intermolecular distance can be calculated (B).
(TIF)

Figure S4 **Persistence length analysis.** Left: Typical force extension curve during bond breakage of E1-5 constructs (green curve). A WLC-fit (blue) provides a persistence length of 0.5 nm, consistent with the stretching of a single polypeptide chain. Right: Persistence lengths obtained from WLC-fitting of rupture events recorded at different pulling speeds.
(TIF)
