# Supplementary figures and images for: Covalent and Density-Controlled Surface Immobilization of E-Cadherin for Adhesion Force Spectroscopy
Source: PLoS One. 2014 Mar 27;9(3):e93123. doi: 10.1371/journal.pone.0093123 (PMC3968077; doi:10.1371/journal.pone.0093123)

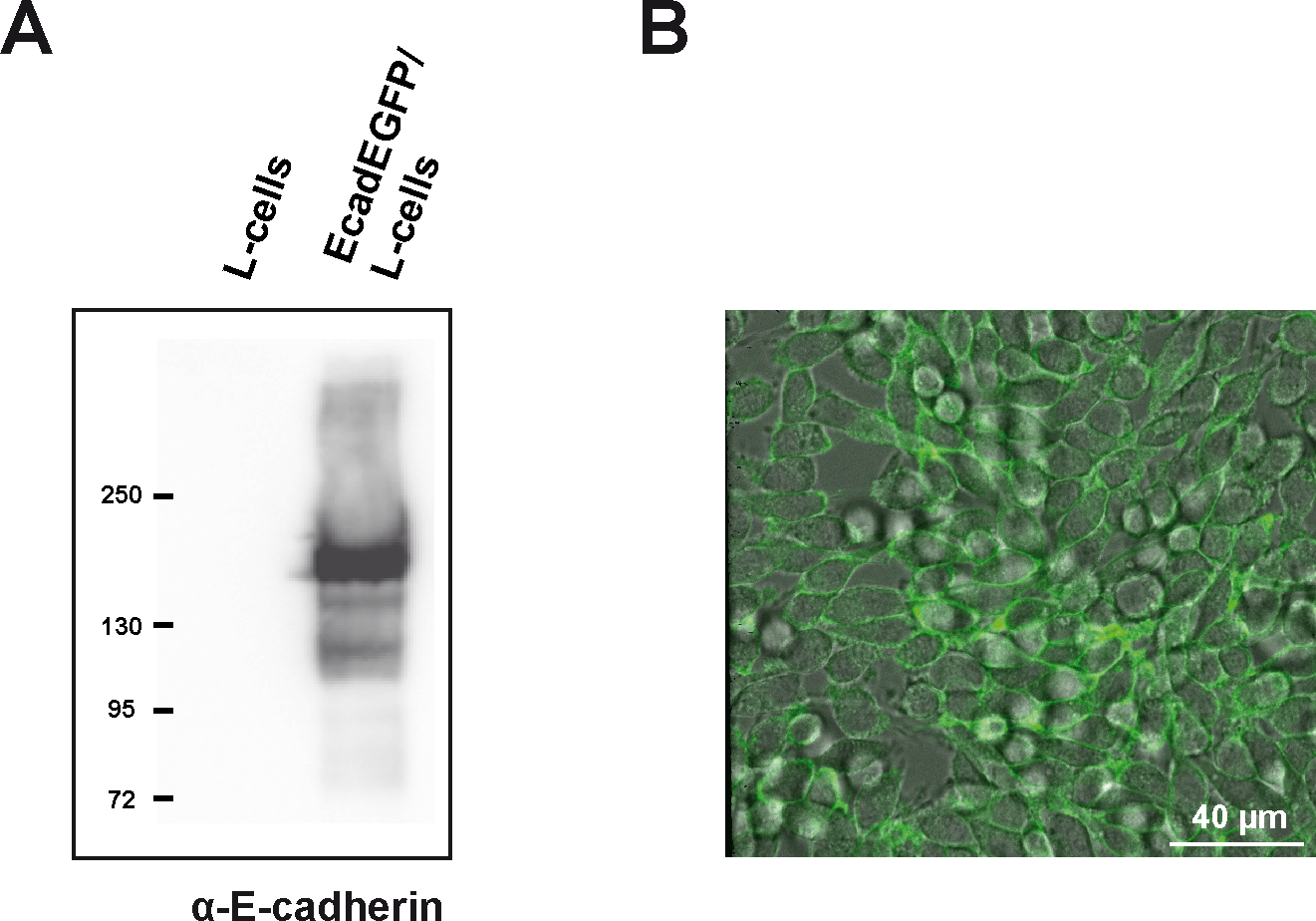

Supplement: Figure S1 — Western-blot analysis of L-cells. (A) Western-blot analysis of wildtype L-cells and EcadEGFP expressing L-cells. Merged phase contrast and fluorescence images of EcadEGFP/L-cells (B). Junctional localization of EcadEGFP indicates proper cell adhesion function of the construct in L-cells. (TIF) [file pone.0093123.s001.tif]

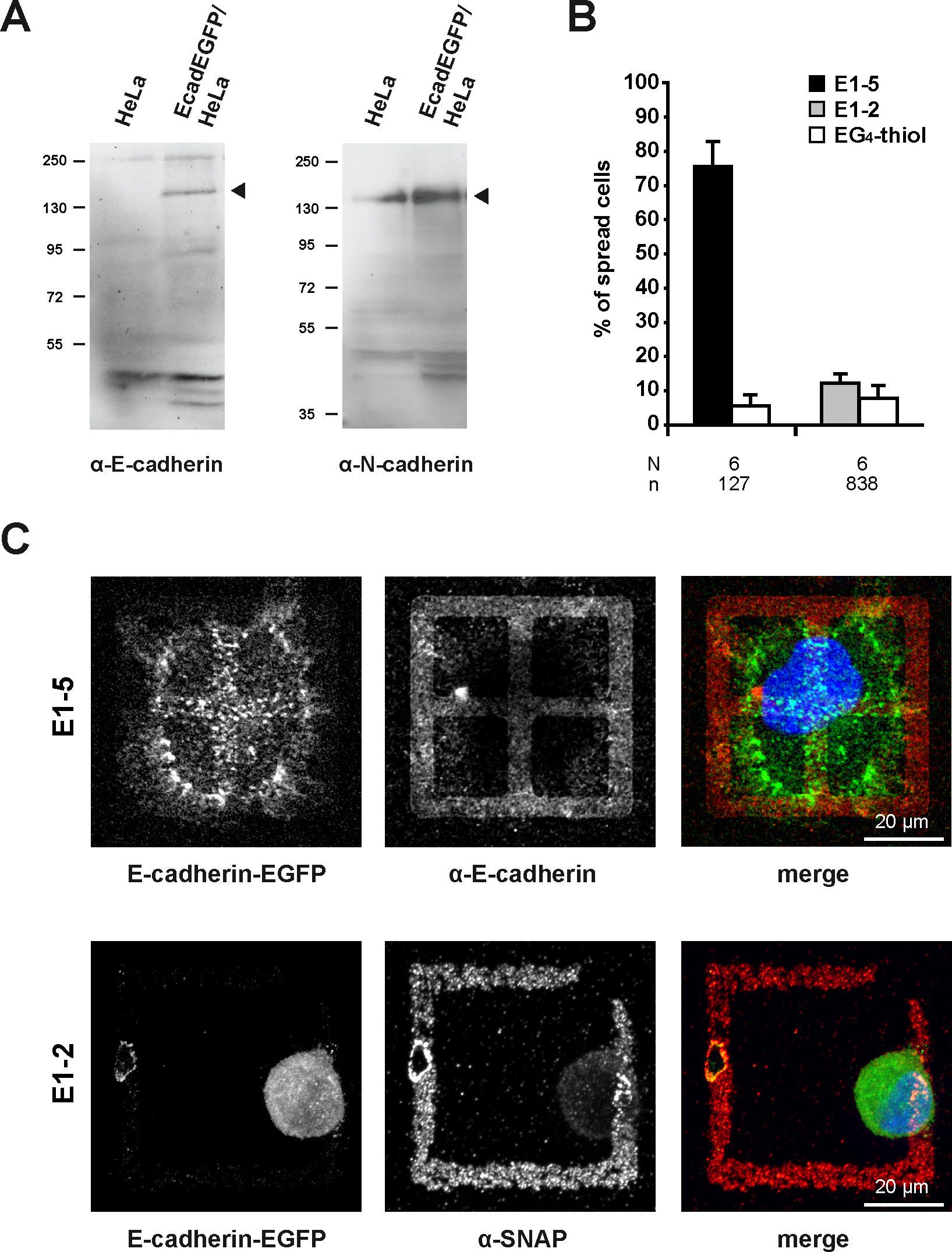

Supplement: Figure S2 — Western-blot analysis of HeLa cells. (A) Western-blot analysis of wildtype HeLa cells and EcadEGFP-transfected HeLa cells for E-cadherin (right panel) or N-cadherin (left panel). Statistical analysis of EcadEGFP/HeLa cells spread on E1-5 or E1-2 patterns or on EG4-thiol. N: numbers of experiments, n: numbers of cells, standard error is shown (B). Fluorescence image of EcadEGFP/HeLa cells on microcontact printed surfaces functionalized with E1-5 or E1-2. Green (C): EcadEGFP fluorescence, red: immunostaining against E-cadherin (E1-5) or SNAP-tag (E1-2), merge: overlay with nuclei staining (DAPI). (TIF) [file pone.0093123.s002.tif]

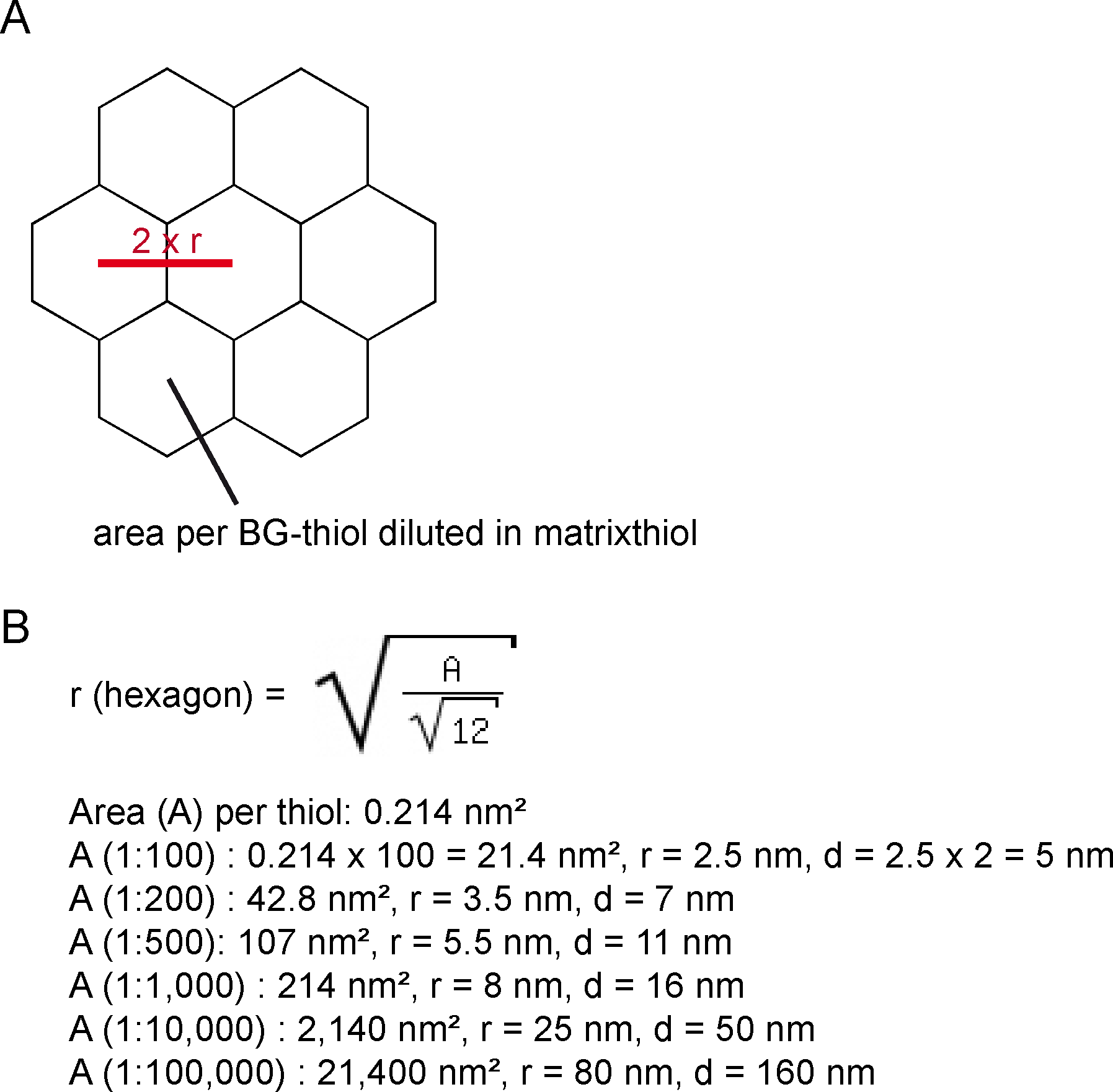

Supplement: Figure S3 — Average distance calculation of E-cadherin monomers. (A) Theoretical hexagon grid used for the calculations. Each hexagon marks the area occupied by one benzylguanine thiol diluted in matrixthiol. The intermolecular distance d corresponds to 2× the radius r of the hexagon. Based on Harder et al. 1998, the area A occupied per thiolate is 0.214 nm2 [34]. When this value is multiplied with the dilution ratio, the hexagon radius and the intermolecular distance can be calculated (B). (TIF) [file pone.0093123.s003.tif]

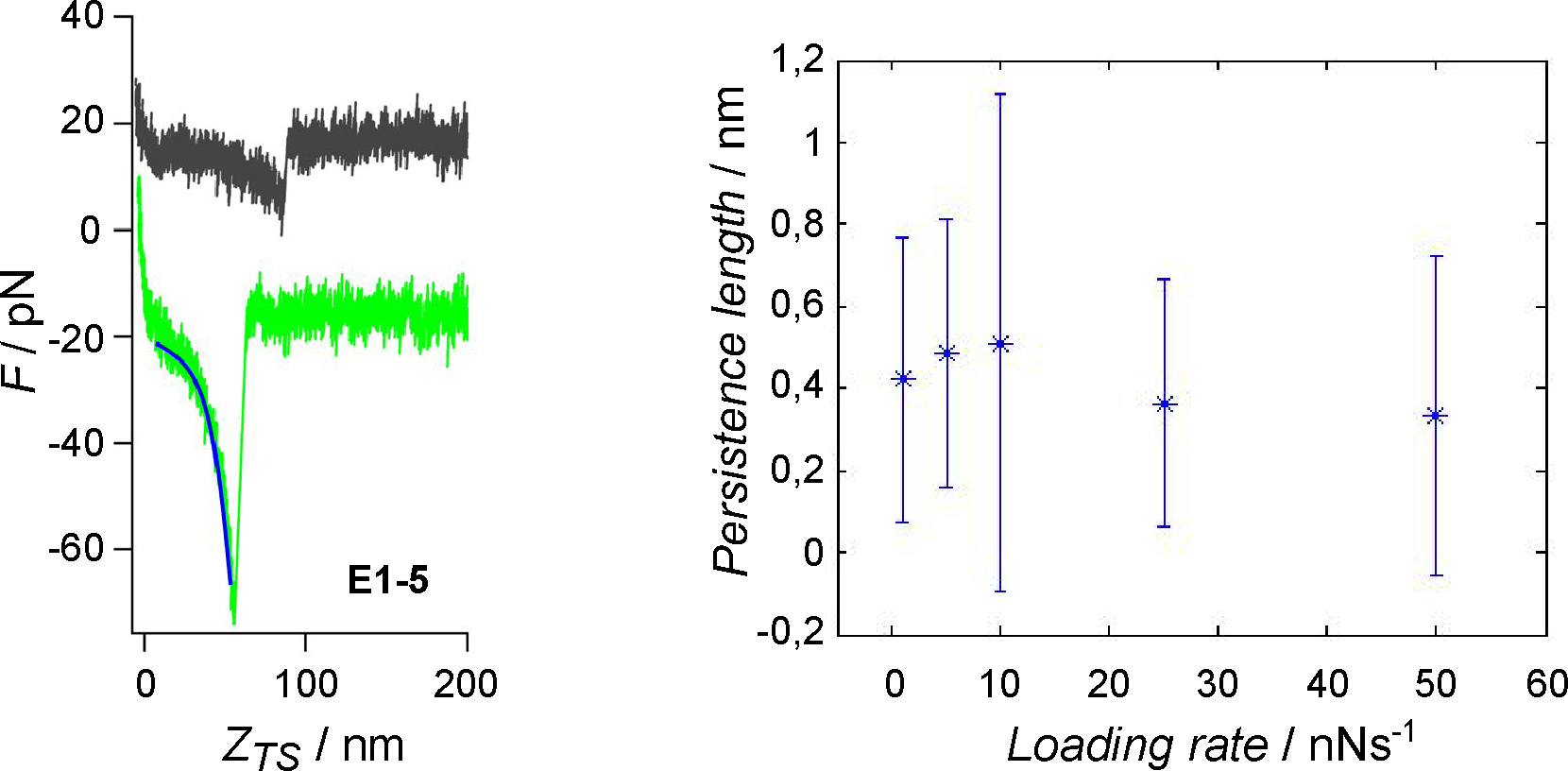

Supplement: Figure S4 — Persistence length analysis. Left: Typical force extension curve during bond breakage of E1-5 constructs (green curve). A WLC-fit (blue) provides a persistence length of 0.5 nm, consistent with the stretching of a single polypeptide chain. Right: Persistence lengths obtained from WLC-fitting of rupture events recorded at different pulling speeds. (TIF) [file pone.0093123.s004.tif]
